# Real-world deployment of remote sleep monitoring technologies reveals distinct patterns associated with cognitive decline

**DOI:** 10.1038/s41746-026-02964-0

**Published:** 2026-07-21

**Authors:** Nan Fletcher-Lloyd, Nathalia Céspedes Gómez, Alexander Capstick, Antigone Fogel, Marirena Bafaloukou, Mahan Heydari, Alexandra Cairns, Chloe Walsh, Jessica True, CR T Group, Nan Fletcher-Lloyd, Nathalia Céspedes Gómez, Alexander Capstick, Antigone Fogel, Mahan Heydari, Alexandra Cairns, Chloe Walsh, Jessica True, Ramin Nilforooshan, Payam Barnaghi, Behnam Shariati, Ramin Nilforooshan, Payam Barnaghi

**Affiliations:** 1 Department of Brain Sciences, Imperial College London, London, UK; 2 UK Dementia Research Institute, Care Research and Technology Centre, London, UK; 3 UK Dementia Research Institute, Imperial College London, London, UK; 4 Surrey and Borders Partnership NHS Foundation Trust, Leatherhead, UK; 5 University College London, London, UK; 6 Great Ormond Street Hospital NHS Foundation Trust, London, UK

**Keywords:** Biomarkers, Diseases, Health care, Medical research, Neurology, Neuroscience, Risk factors

## Abstract

Examining sleep patterns in relation to chronological ageing and dementia can provide insights for risk screening. Integrating predictive models with remote sleep monitoring enables routine assessment of cognitive decline symptoms in high-risk groups, aiding early risk identification. We developed a machine learning pipeline to estimate Sleep Age Index from longitudinal under-the-mattress sleep sensor data in the general population and a dementia cohort (*n* = 1672; person-samples = 18,369), using it to identify dementia risk. Risk scores were stratified into high, medium and low-risk categories to support clinical decision-making. Our study indicates that sleep patterns in dementia do not follow typical ageing processes, with the pre-trained model showing greater deviation from “normative” age-related patterns. These deviations were associated with irregular bed- and rise-times and reduced night-to-night variability in deep sleep. Chronological age was predicted from sleep data with a mean absolute error of 5.52 (95% CI: 5.37–5.67) on held-out data. In dementia versus control, the model achieved 75.7% (95% CI: 71.4%–79.9%) sensitivity and 74.7% (95% CI: 69.2%–80.0%) specificity post-stratification on unseen data. In a pilot high-risk cohort (*n* = 50), model predictions showed slight positive bias relative to clinical judgement (mean difference 0.98, limits of agreement −0.83–2.78). These findings demonstrate the potential of remote sleep monitoring and predictive modelling in identifying individuals who may benefit from further clinical evaluation and early intervention.

## Introduction

There are approximately 57 million people living with dementia (PLWD) worldwide^1^. It is estimated that three-quarters of individuals with dementia have not received a formal diagnosis, which significantly limits their access to treatment, care, and support^2^. In low- and middle-income countries, this “treatment gap” is even more pronounced, reaching as high as 90%^2^. As a consequence, people living with undiagnosed dementia are more likely to require emergency care and hospitalisations than their age-matched healthy counterparts^3–5^. Despite this, dementia remains underdiagnosed across the globe, particularly in the earlier stages of its progression, where symptoms may instead be considered congruent with cognitive ageing^2^. Even in the absence of disease-modifying treatment, early risk stratification for dementia has positive implications for managing comorbidities, mitigating safety risks, preventing complications, and supporting patients and their caregivers in planning for the future. Current screening methods, such as Positron Emission Tomography (PET), Magnetic Resonance Imaging (MRI) scans and fluid biomarker tests, are expensive, invasive, and unsuitable for large-scale screening. As such, there is an unmet need for more affordable, safe, and non-invasive tools that can reliably screen for dementia in the general population.

Age is a significant risk factor for dementia, with incidence increasing exponentially in older populations^6^. Aging is also associated with significant changes in sleep patterns, including shorter, more fragmented sleep, with increased nocturnal sleep disturbances^7–11^. Moreover, older adults are more likely to experience multi-morbidity (the co-existence of two or more chronic conditions^12^), with more than half of adults over the age of 60 having multi-morbidity^13,14^. Multi-morbidity in older adults is also associated with self-reported sleep problems^15–17^, which are often considered an outcome of multimorbidity but are also associated with a higher risk of experiencing symptoms of various health conditions^18^. In particular, changes in sleep physiology are also associated with an increased risk of dementia^19,20^, and dementia-related brain pathology often manifests as disrupted sleep patterns emerging in the early stages of the disease and continuing throughout its progression^21–23^. However, the exact causal relationship between sleep and cognitive decline remains unclear.

Recent works have used both electroencephalography (EEG) and polysomnography (PSG)-derived sleep data to predict age^24–29^. Considering the association between age and changes in sleep patterns, predicted sleep has been proposed as a potential marker related to brain health^29^. Sleep Age Index (SAI) is measured as the difference between a person’s chronological and predicted age^24–29^. Higher SAIs have been statistically associated with a reduced life expectancy^26,29^, as well as an increased risk of a variety of health conditions, including diabetes^25^, HIV^28^, hypertension^25^, and dementia^27^. Despite this, it remains unclear whether SAI captures clinically meaningful deviations that can stratify dementia risk.

While these works have leveraged either EEG or PSG-derived sleep data, no studies have yet exploited remote monitoring of sleep in real-world settings to estimate age. PSG, a comprehensive assessment of sleep physiology, includes EEG, electrocardiography (ECG), electrooculography (EOG), chin and leg electromyography (EMG), and assessments of breathing effort and airflow^29^. While PSG is widely regarded as the gold standard for diagnosing sleep disorders, its high running costs, intensive administrative requirements, and limited accessibility significantly constrain its capacity to generate broad clinical impact^29,30^. Sleep EEG offers a more cost-effective and simpler alternative to PSG, but shares many of the limitations of using PSG, one of the main ones being how the unfamiliar environment required for data collection can alter participants’ typical sleep patterns^31^. The advent of remote monitoring technologies has extended our ability to study sleep. Unlike PSG and EEG, remote monitoring technologies such as wearable devices and under-the-mattress sleep sensors^30^ enable the collection of sleep data in individuals’ natural sleep environments. However, wearable technologies usually have a short battery life, requiring regular charging and frequent removal^32^. In studies involving populations where memory or dexterity may be impaired, this can limit the reliability of longitudinal monitoring. For this reason, we sought to assess the feasibility of using under-the-mattress sensors to study changes in sleep patterns associated with age and dementia.

**Fig. 1:**
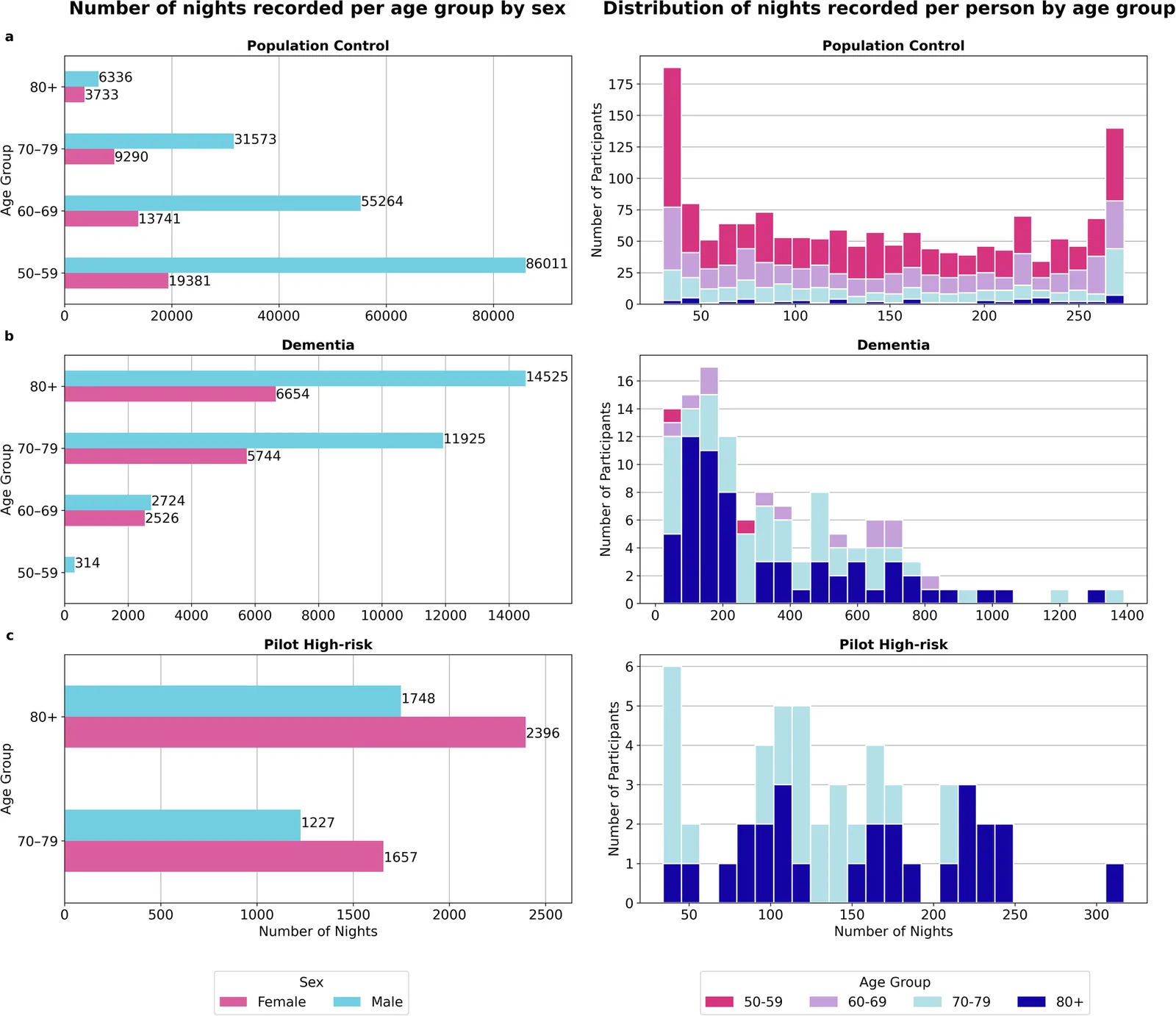
The composition of the pre-processed datasets from which the final processed samples were derived. The total number of nights recorded across age and sex (left-hand column), and the distribution of nights recorded by participant (right-hand column), where each bar represents the number of participants for which a given number of nights were recorded, illustrating the variability in counts across participants, and counts are grouped into 25 bins. Participants were grouped into four age bands. Coloured bars represent the four different age groups: 50–59, 60–69, 70–79, and 80+. **a** Population Control cohort. **b** Dementia cohort. **c** Pilot High-risk cohort. Figure created with Matplotlib and Seaborn.

This study presents a two-stage machine learning approach to identify dementia risk from SAI. The SAI scores are predicted using in-home nocturnal activity and physiology data collected by under-the-mattress sleep sensors. We have also developed a risk stratification method to improve the clinical applicability of our framework. The models have been trained and validated using two datasets: (1) a dataset comprised of 1567 individuals from the general population, used here as a control, and (2) a dataset comprised of 105 individuals participating in an ongoing clinical study observing PLWD in their own homes. We also assessed the utility of our work as a tool to identify dementia risk by applying our approach to a high-risk population of 50 individuals in a pilot study. Figure 1 provides an overview of the three pre-processed datasets used in the training and evaluation of this approach. Our work (1) offers insights into how sleep changes with age, multimorbidity, and dementia, providing a foundation to guide discovery research (2) offers an early risk identification method and clinical decision support for earlier risk identification of dementia.

## Results

This work included nocturnal activity and physiology data from three cohorts collected using under-the-mattress sleep sensors. After data processing, the Population Control cohort was comprised of 15,229 samples, collected from 1567 participants (326 female, 1241 male), the Dementia cohort was comprised of 3140 samples collected from 105 participants (40 female, 65 male), and the Pilot High-risk cohort was comprised of samples 470 collected from 50 participants and their study partners (40 participants, 10 study partners; and 29 female, 21 male). Table 1 reports the demographics of the three datasets before and after data processing.

**Table 1:** Cohort demographics

Cohort	Dataset	Sample size, n	Sex, (Female, Male)	Age range
**Population Control**	Original	1,080,081	F = 1034, M = 4540	19–99
**Population Control**	Processed	15,229	F = 326, M = 1241	50–98
**Dementia**	Original	116,862	F = 82, M = 78	51–100
**Dementia**	Processed	3140	F = 40, M = 65	52–92
**Pilot High-risk**	Original	14,412	F = 44, M = 30	72–98
**Pilot High-risk**	Processed	470	F = 29, M = 21	72–92

## Age estimation

We trained a regression model to estimate age using a training set comprised of 12,138 samples, collected from 1253 participants (257 female, 996 male) derived from the Population Control cohort. We evaluated and compared the effectiveness of our model using three different regression models: Elastic Net, Huber, and Extreme Gradient Boosting (XGBoost). Table S10 in Supplementary Information Section S8 shows the results of the 10-fold cross-validation on all models tested. We compared model performance with and without sample weighting. We found the best-performing regression model was XGBoost with *L*1 regularisation, without sample weighting. While sample weighting tended to marginally improve performance on under-represented ages (as measured by the balanced mean absolute error, or BMAE) across all models, the overall regression performance (as measured by mean absolute error, or MAE, mean squared error, or MSE, and root mean squared error, or RMSE) was almost always slightly worse compared to the unweighted model. Table 2 presents the model’s performance on both the validation data (averaged across the 10-fold cross-validation) and on the held-out test data (definitions of evaluation metrics can be found in Supplementary Information Section S3).

**Table 2:** Mean (95% CI) % of MAE, MSE, RMSE, and BMAE of the age estimation model on the different data splits

	MAE	MSE	RMSE	BMAE
**Validation**	5.97 (5.73–6.19)	56.0 (52.0–59.7)	7.47 (7.20–7.72)	6.59 (6.29–6.87)
**Test**	5.52 (5.37–5.67)	48.9 (46.2–51.6)	6.99 (6.80–7.17)	7.37 (7.06–7.69)

*MAE* Mean Absolute Error, *MSE* Mean Squared Error, *RMSE* Root Mean Squared Error, *BMAE* Balanced Mean Absolute Error.

## How sleep metrics contribute to age prediction

**Fig. 2:**
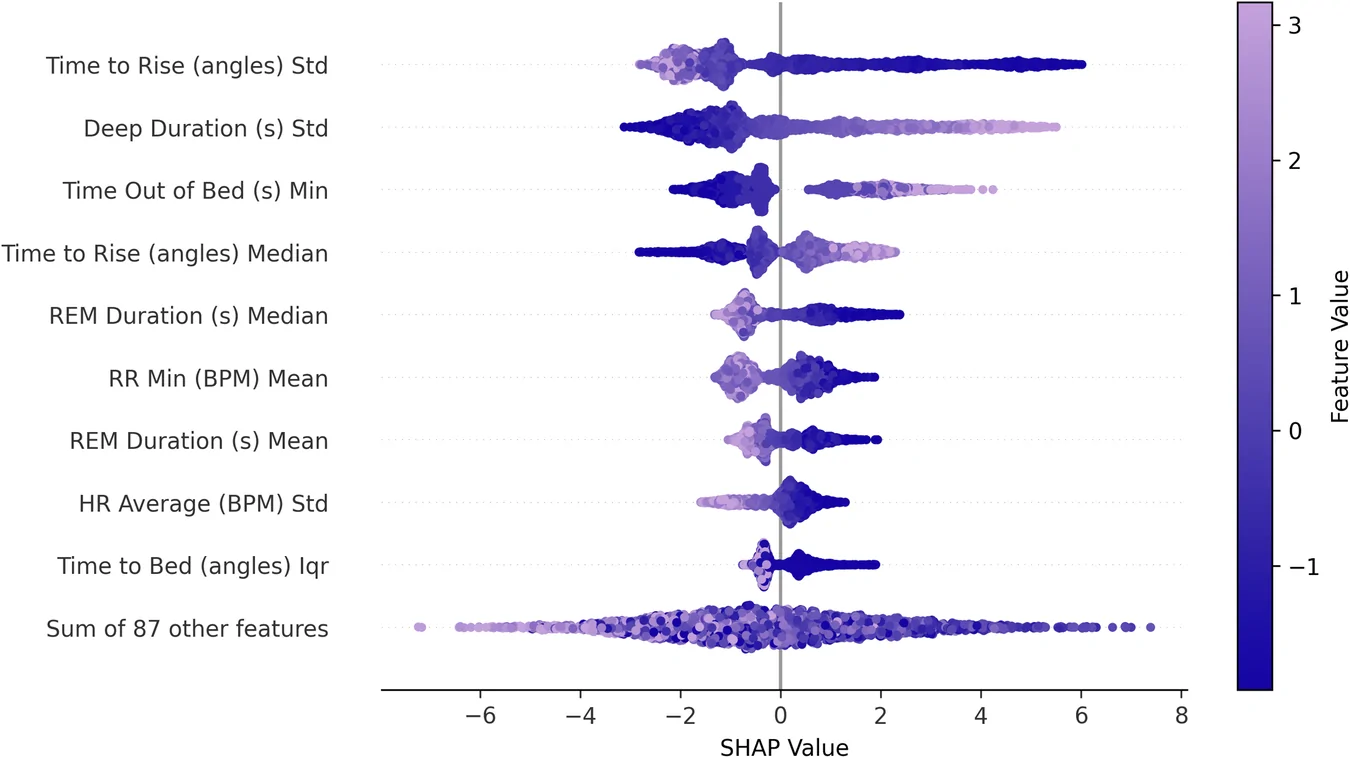
SHAP values. The feature importance for the top 10 most important features, as calculated on the held-out test set using SHAP. The corresponding feature values are coloured according to the standardised value of that feature (high values shown in lilac and low values in dark blue). The position along the x-axis shows the SHAP value, representing the magnitude and direction of each feature’s contribution to the prediction. Positive values push the prediction higher, while negative values push it lower, relative to the model’s baseline (average) prediction (denoted by 0 on the x-axis). Figure created with Matplotlib, Seaborn, and SHAP.

SHapley Additive exPlanations (SHAP) were used to explain the individual predictions of the model. In regression tasks, SHAP plots show how much each feature contributes to the prediction for a given instance above or below the baseline (average predicted value, in this case, age). The magnitude of a SHAP value indicates how strongly a feature impacts a specific prediction, while the sign (positive or negative) indicates whether the feature increases or decreases the predicted age compared to the baseline, respectively. This demonstrates the key sleep metrics contributing to age prediction. Figure 2 shows a summary plot of SHAP values for the held-out test set from the Population Control cohort. The features are ordered with respect to their global importance. Figure S4 in Supplementary Information Section S7 shows a summary of the key sleep metrics contributing to age prediction in the Dementia cohort.

## Calculating SAI

To calculate the SAI, we applied a three-step procedure (see Fig. S1 in Supplementary Section S1 for a visualisation). In Step 1, we obtain predicted sleep ages for each cohort (Population Control test set, Dementia, and Pilot High-risk) using our pre-trained age estimation model. The pre-trained model used the control dataset for training and validation. We compared the performance of our age estimation model across female and male participants, within both the Population Control and Dementia cohorts (see Table S9 in Supplementary Information Section S6). In Step 2, we adjust these predictions based on age-group-weighted mean differences between the sleep and chronological age in the Population Control test set (*n* = 314 participants, 69 female, 245 male). For model training and hyperparameter tuning, we used broader age bands (50-59, 60-69, 70-79, and 80+) for stratification and sample weighting to maintain training stability and reduce data fragmentation. Narrower age bands (50-54, 55-59, 60-64, 65-69, 70-74, 75-79, and 80+) were then used to calculate age-group-weighted means (as in step 2) to better capture the clinically observed age-related changes in sleep patterns. These means were then subtracted from the predicted sleep ages (computed in step 1) across all three datasets, based on the participant’s age group. In step 3, we computed the SAI for each participant as the difference between the age-group-adjusted sleep age and chronological age.

A statistically significant difference in SAI means was observed between the Dementia cohort (*n* = 3140, mean = −4.21, SD = 5.86) and the Population Control cohort (*n* = 3091, mean = 1.34, SD = 4.55) (*t* = −41.7, *p* < 0.001), all reported to 3.s.f., supporting the use of SAI as a predictive feature in our dementia risk screening model. Figure S5 in Supplementary Information Section S7 visualises the results of a quantitative analysis comparing SAI with severity of cognitive impairment for individuals in the Dementia cohort. Severity classifications follow the guidelines of the National Institute for Health and Care Excellence^33^ (see Table S2 in Supplementary Information Section S2). Interestingly, a U-shaped relationship is observed, with SAI being lower in participants with mild, moderate, and moderately severe dementia compared to those with MMSE ≥27, but higher in participants with severe dementia. These patterns reflect heterogeneity in how sleep features relate to cognitive decline across disease stages, suggesting that the model’s SAI does not increase monotonically with disease severity.

## Dementia risk prediction

We trained and evaluated a binary classification task to predict dementia risk by differentiating between the SAI of an individual from the Dementia cohort from that of an individual from the Population Control cohort. In total, our dataset consisted of 6231 samples, collected from 419 participants (109 female, 310 male). Our training set was comprised of 4927 samples, collected from 334 participants (79 female, 255 male). We evaluated and compared the effectiveness of two different classification models: Logistic Regression and XGBoost. Table S10 in Supplementary Information Section S8 shows the results of the 10-fold cross-validation on all models tested. We also compared model performance with and without sample weighting. We found the best-performing classification model was Logistic Regression with *L*2 regularisation, without sample weighting. Table 3 presents the model’s performance on both the validation data (averaged across the 10-fold cross-validation) and on the held-out test data (definitions of evaluation metrics can be found in Supplementary Information Section S3).

**Table 3:** Mean (95% CI) % of sensitivity, specificity, and area under the precision-recall curve of the dementia risk prediction model on the different data splits

Before Stratification			
	Sensitivity	Specificity	AUC Precision-Recall
**Validation**	68.8 (61.2–75.9)	71.5 (69.0–73.8)	78.8 (71.7–85.3)
**Test**	69.2 (65.5–72.7)	62.8 (59.1–66.5)	76.8 (73.7–79.7)
**After Stratification**			
	Sensitivity	Specificity	Precision
**Validation**	79.9 (68.7–91.0)	86.7 (76.1–93.7)	86.7 (74.7–94.9)
**Test**	75.7 (71.4–79.9)	74.7 (69.2–80.0)	82.1 (78.0–86.1)

These results are reported before and after stratification is performed.

## Risk stratification

**Fig. 3:**
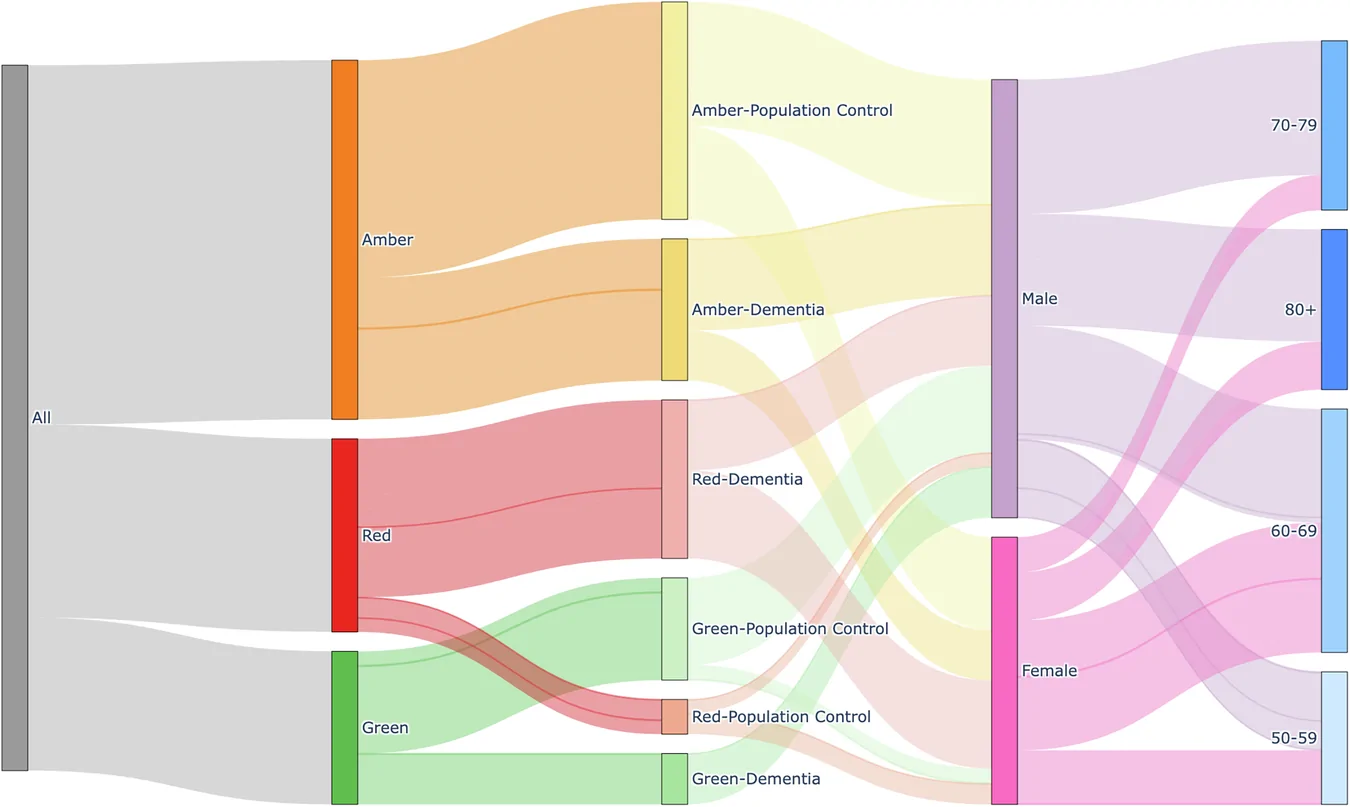
Sankey diagram showing stratification breakdown of risk prediction model. Analysis of stratified groupings produced by evaluating the final classification model on the held-out test set using optimised thresholds. `Green' represents the samples that the model confidently predicts are from an individual in the Population Control cohort, `Amber' represents the samples that the model is not confidently predicting, and `Red' represents the samples that the model confidently predicts are from an individual in the Dementia cohort. Each group is further broken down by the true labels, then by the sex of the participant from which the sample came, and finally by age (using the four age bands for the sake of interpretability). View an interactive version of the figure here: https://tmi-lab.github.io/Sleep-Age-Dementia/sankey_diagram.html. Figure created with Matplotlib.

We developed a traffic light system to stratify the dementia risk based on the model’s confidence in its predictions. We varied stratification thresholds to balance sensitivity and specificity with the number of samples stratified into the Red and Green groups. In this work, we select thresholds [0%, 30%], (30%, 70%], and (70%, 100%] (following interval notation) for the ‘Green’, ‘Amber’, and ‘Red’ groups, respectively. Table 3 shows the model performance after stratification when evaluating the predictions on the ‘Green’ and ‘Red’ groups. Figure 3 reports the distribution of the model’s predictions by group (including the ‘Amber’ group) on the held-out test set (*n* = 1304, *p* = 85). We provide an analysis of model reliability and calibration in Supplementary Information Fig. S6 in Supplementary Information Section S9. We also compare model performance between sex and across age groups (see Supplementary Information Tables S11 and S12, respectively, in Supplementary Information Section S11), as well as the diagnostic value of SAI (see Supplementary Information Tables S13 and S14, respectively).

## Pilot study in a high-risk population

To assess the predictive accuracy of our framework, we ran a pilot study within the NHS. We applied our classification model (and stratification thresholds) to the SAI for the Pilot High-risk cohort, a real-world high-risk population of individuals who may plausibly have undiagnosed dementia or go on to develop the condition.

Following dementia risk prediction on the held-out test set (*n* = 1304, *p* = 85), we calculated the age-group weighted mean dementia risk score using the narrower age bands (50–54, 55–59, 60–64, 65–69, 70–74, 75–79, 80+) for all known positive cases of dementia. We then subtracted these values from the dementia risk scores predicted for each sample in the Pilot High-risk cohort dataset (n=470, p=50). This allowed us to estimate similarity to diseased individuals by looking at how closely the dementia risk score of an individual in the Pilot High-risk cohort looks compared to the dementia risk scores of their age-matched counterparts in the Dementia cohort. By controlling for age, we are also able to correct for demographic data drift.

Using the age-group-adjusted difference between dementia risk scores, alongside stratification, we reassigned participant samples into three groups: ‘Red’, ‘Amber’, and ‘Green’, which represent ‘Highly Likely’, ‘Somewhat Likely’, and ‘Not Likely’ risks of cognitive decline, respectively. Samples were reassigned using the following rules:

- If group == 'Red' and diff ≥ 0, return 'Highly Likely'
- If group == 'Red' and diff < 0 or group == 'Amber' and diff ≥ 0, return 'Somewhat Likely'
- Otherwise, where group == 'Amber' and diff < 0 or group == 'Green', return 'Not Likely'

**Fig. 4:**
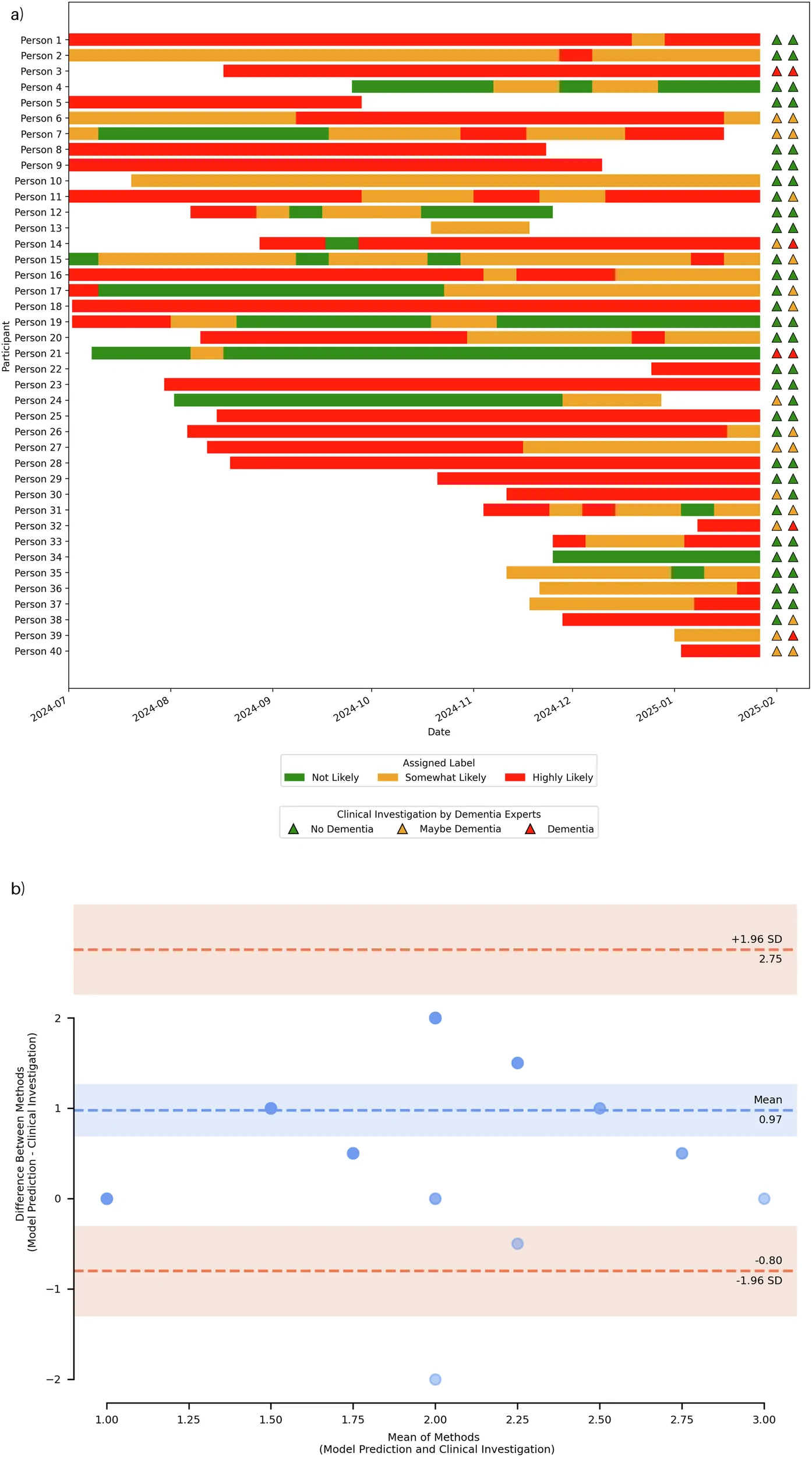
Model predictions and agreement with expert annotations across the Pilot High-risk cohort. **a** Dementia risk over time for individuals in the Pilot High-risk cohort. Bar colours correspond to the predicted likelihood that an individual might have dementia (the assigned label): 'Not Likely' is shown in green, 'Somewhat Likely' in amber, and 'Highly Likely' in red. Triangle colours correspond to the clinical label given to each participant during re-assessment: 'No Dementia' is shown in green, 'Maybe Dementia' in amber, and 'Dementia' in red. Each triangle represents the clinical ground truth determined by a single expert. Figure created with Matplotlib and Seaborn. **b** Bland-Altman plot showing agreement between the model’s predictions and expert annotations. Each point represents a paired measurement. From the 40 participants, we derive 11 unique paired measurements. The mean difference (bias) is represented by a dashed blue line, while the lower and upper limits of agreement (mean difference ± 1.96 SD) are represented by dashed red lines. Figure created with Matplotlib and pyCompare.

On applying these criteria for reassignment to our Pilot High-risk cohort, we were able to generate profiles for each participant based on the model’s confidence that they may be experiencing cognitive decline over time. To evaluate the utility of our pipeline as a tool for identifying dementia risk, a subset of this cohort (the study participants, *n* = 40) underwent clinical assessment. Two clinicians (experts in dementia) received a list of the participant IDs included in our analysis, but were blinded to the model’s output to reduce potential bias. We measured the agreement between the predictions for each participant and the ground truth determined by clinical investigation. For the clinical investigation, the experts used the Addenbrooke’s Cognitive Examination-III (ACE-III) scores and electronic health records of each participant in the pilot study. Figure 4a shows how dementia risk as predicted by our model varies over time in individuals from the high-risk population. We have coloured the bars by the stratification groups to which participants were reassigned based on their adjusted risk scores. To further protect individual privacy, we replaced the original anonymous participant IDs with a new set of randomly generated numbers. We have further annotated the plot with the clinical labels given by each clinician (an expert in dementia) to each participant during re-assessment. Figure 4b is a Bland-Altman^34^ plot visualising the level of agreement between the model’s predictions and expert annotations. The pilot data analysis indicated a slight positive bias between the model’s predictions and the clinical ground truth (mean difference 0.98 units, with limits of agreement from -0.83 to 2.78 units (mean difference ± 1.96 SD), all reported to 3.s.f.).

Finally, after accounting for within-participant variability using linear mixed-effects modelling, no statistically significant association was found between the adjusted risk scores and ACE-III scores (*β* = 0.05, sd = 0.03, *z* = 1.49, *p* = 0.14), reported to 3.s.f. Moreover, no statistically significant association was found between either the adjusted risk scores and PHQ-9 scores (*β* = −0.05, sd = 0.06, *z* = −0.94, *p* = 0.35) or the GAD-7 scores (*β* = −0.03, sd = 0.05, z = −0.60, *p* = 0.55), respectively, reported to 3.s.f.

## Discussion

Sleep patterns undergo significant alterations with age. These alterations are reflective of changes in the broader neurological and physiological processes that occur during ageing. In recent years, the concept of leveraging sleep age, a measure derived by estimating chronological age using sleep features, has emerged as a promising data-driven index by which to measure brain health^24–29^. An increase in predicted sleep age compared to chronological age has been associated with increased mortality and morbidity, including neurodegenerative conditions and cognitive decline, suggesting its potential in monitoring and early disease detection^25–29^.

In this study, we derived a Sleep Age Index (SAI), defined as the deviation between predicted and chronological age, and examined whether such deviations are associated with dementia. Importantly, it should be noted that SAI is not intended as a measure of biological ageing, but rather as a data-driven marker derived from a model trained to predict chronological age in a predominantly healthy population. It measures deviations in sleep patterns between people living with dementia and the general population, which can be used to identify differences associated with dementia.

Previously, we published a paper comparing sleep patterns between people living with dementia and the general population^35^. In this study, our objective was not to optimise prediction of cognitive impairment, but to derive a continuous, interpretable deviation metric, which is a clinically available and verifiable variable. Our primary aim was to explore whether SAI derived from remote sleep monitoring carries clinically relevant information about cognitive status and disease progression that may assist dementia risk stratification. By using explainable machine learning models with traceable decision-making processes, we aimed to develop an interpretable tool that could complement clinical assessment and decision-making.

We present a two-stage machine learning approach for estimating dementia risk using SAI derived from remote sleep monitoring data. We considered several regression models for age estimation and found that XGBoost attained the top performance (MAE of 5.52 (95% CI: 5.37–5.67), MSE of 48.9 (95% CI: 46.2–51.6), and BMAE of 7.37 (95% CI: 7.06–7.69)). This performance was comparable to other age estimation models trained on EEG and PSG data^24,25^, including those using deep neural networks^29^.

We next considered two common classifiers for age estimation and found that Logistic Regression attained the top performance (sensitivity of 69.2% (95% CI: 65.5%–72.7%) and specificity of 62.8% (95% CI: 59.1%–66.5%). The ratio of positives (samples from individuals in the Dementia cohort) to negatives (samples from individuals in the Population Control cohort) in the test set is 1 : 1.01. We then transformed dementia risk scores into three tuneable groups, allowing us flexibility in the management of model accuracy and uncertainty. Post-stratification, the performance on the ‘Green’ and ‘Red’ groups was improved, achieving a sensitivity of 75.7% (95% CI: 71.4%–79.9%), and a specificity of 74.7% (95% CI: 69.2%–80.0%) (see Supplementary Information Section S2 for definitions of evaluation metrics). Grouping the classification model’s predictions based on the dementia risk score also increased accuracy in each sex and across the four age bands, as shown in Tables S11 and S12 in Supplementary Information Section S11. A breakdown of these predictions can be seen in Fig. 3.

We conducted a Pilot study applying our model to a high-risk population of multi-morbid individuals. Figure 4b visualises an analysis of the agreement between the model’s predictions and the ground truths established by dementia experts. This analysis indicated a slightly positive bias (0.98) overall and moderate agreement between the model and expert annotations. Specifically, there was good agreement in clear cases of dementia or no dementia, but slightly poorer agreement for more ambiguous cases. These findings suggest that our model is reliable for distinguishing between PLWD and those without, but somewhat less reliable for borderline cases. We also present an evaluation of the relationship between the adjusted risk scores and the ACE-III, the Patient Health Questionnaire-9 (PHQ-9) and Generalised Anxiety Disorder Questionnaire-7 (GAD-7) scores, respectively, in the Pilot High-risk cohort. Accounting for within-subject variability, linear mixed-effects modelling found no statistically significant associations across all three pairings. Self-reporting can introduce subjectivity and potential inaccuracies in responses^36^. This bias can compromise data reliability and this may contribute to the lack of statistically significant associations observed in this study. Moreover, the absence of correlation between SAI and these scores may reflect several factors beyond sample size limitations. SAI has been shown to exhibit a non-linear, U-shaped relationship with cognitive severity in the Dementia cohort. This non-linearity would inherently limit correlation with a scalar clinical measure. Furthermore, measures such as ACE-III, PHQ-9, and GAD-7, while clinically useful, provide an episodic and broad assessment of cognitive domains that may lack the granularity to reflect the discrete neurophysiological processes indexed by SAI. As such, the absence of correlation with these measures should not be interpreted as evidence against SAI’s potential as an interpretable data-driven marker to support dementia risk stratification. Individuals in the Dementia cohort who were not detected by our model had a higher mean MMSE than those who were correctly identified, suggesting SAI may lose sensitivity at earlier stages of cognitive impairment. However, given the small sample size, these findings should be considered exploratory and the relationship between SAI sensitivity and disease severity warrants further investigation in larger cohorts. Overall, these findings serve as preliminary evidence of the feasibility for use of our framework as a risk screening tool in larger clinical trials. Subsequent analysis also reveals that the traffic light system, designed to indicate the risk of cognitive decline, can serve as a complementary source of information to enhance decision support, which is especially useful for high-risk groups, where there is often insufficient information regarding an individual’s cognitive status.

Our approach uses SHAP analysis to reveal how individual sleep metrics contributed to age prediction. Based on analysis of held-out test data from the Population Control cohort, we observe that higher variability of time to bed and time to rise were associated with lower predicted ages. This aligns with previous findings showing that the sleep-wake schedules of adults < 65 years of age tend to be more irregular, impacted by changing social and working schedules that make it harder to maintain a consistent sleep routine compared to adults > 65 years of age^37^. Likewise, higher variation in heart rate and higher minimum respiratory rate are also associated with lower age predictions. The latter suggests that low respiratory rates contribute to higher age predictions. Our analysis also highlighted that high variability in deep sleep duration and less REM sleep were associated with higher predicted ages. In particular, disruptions in slow-wave (deep) sleep have been linked to the pathophysiology seen in neurodegenerative disease and have been suggested as a potential biomarker for early diagnosis^38–40^. Finally, more time spent out of bed between going to bed and rising was also associated with higher predicted age, suggesting older adults were experiencing more frequent awakenings. This finding is in agreement with the literature^41^.

In contrast to previous reports^27^, we found PLWD tended to be associated with negative SAI after adjusting for age, whereas positive SAI scores were more commonly observed in the Population Control cohort. SAI reflects deviation from “normative” sleep patterns learned in the general population training distribution and is not calibrated for clinical populations with pathology-related sleep abnormalities. When applied to a cohort with a neurodegenerative condition, the model maps pathological sleep features onto regions of the learned age-feature in latent space. However, this is not intended to reflect shifts or changes in biological age. In other words, the model does not imply that PLWD have sleep patterns indicating age under 65; instead, it reflects model behaviour under distribution shifts and pattern similarities. Importantly, PLWD tended to be associated with greater deviations in magnitude than individuals from the Population cohort. Sleep and ageing are tightly coupled in healthy adults; however, this relationship may be disrupted in neurodegeneration^7^. While dementia is associated with sleep fragmentation and reduced REM and slow-wave sleep^21–23^, these represent pathological changes that do not necessarily align with typical age progression. Our model was trained using a cohort from the general population and was not calibrated for neurodegenerative populations. As such, it has learned sleep patterns associated with typical ageing, explaining our model’s misinterpretation of these abnormal dementia-related patterns with respect to chronological age. For example, longer total sleep time has been associated with poorer cognitive outcomes, frailty and dementia^42,43^, and may drive some of the observed patterns. However, as total sleep time was not directly included as a model input, future work should further consider whether this metric, independent of individual sleep-stage composition, provides additional explanatory value for SAI predictions. Likewise, the fragmentation of sleep in dementia may cause increased restlessness, mimicking the more irregular sleep patterns of adults under the age of 65,^37^ justifying why a PLWD might experience higher time to bed or time to rise variability.

Several strengths and limitations relate to the technology used in this study. We leveraged under-the-mattress sleep sensors to collect personalised longitudinal data from people in the comfort of their own homes. The Withings Sleep Analyzer has been validated to detect total sleep time, sleep efficiency and wake after sleep onset in a range of studies^44–46^, including for people with sleep disorders such as sleep apnoea^47^, and is reported to have comparable performance to wearable sleep trackers^48^. In addition to providing a scalable, low-cost approach for data collection in naturalistic settings (unlike in PSG or EEG studies), under-the-mattress sleep sensors have the potential to reduce data loss compared with wearable devices^48^.

In studies focusing on age-related diseases where individuals may be experiencing cognitive or functional impairment, under-the-mattress sleep sensors boast a particularly important advantage. However, it should be noted that, with respect to sleep staging, the Withings Sleep Analyzer estimates sleep stages indirectly using non-EEG physiological signals from cardiorespiratory and movement signals (see https://support.withings.com/hc/en-us/articles/360000157987). While the proprietary algorithm was calibrated at the Paris-Béclère Hospital against clinical recordings of both sleep and PSG, sleep stages remain inferred rather than directly measured. Therefore, references to REM or deep sleep should be interpreted as proxy-derived estimates rather than direct measures of cortical sleep architecture. Independent validation of sleep-stage performance remains limited. Although a PSG laboratory study by Yang et al. demonstrated accurate estimation of cardio-respiratory metrics during sleep^49^, staging accuracy has been reported as moderate-to-poor (45% to 59%)^48^, which is substantially lower than the accuracy reported for in-home EEG sleep devices (83.9% - 96.4%)^50^.

We also acknowledge that under-bed mattress sensors could, in principle, be influenced by the presence of a bed partner. However, in the Minder and Resilient studies, clinical monitoring teams ensured that the sensor was placed on the participant’s side of the bed to minimise potential interference from co-sleepers. Despite this, information on whether participants routinely slept alone or with a partner was not systematically recorded in the Minder study. Therefore, we are unable to perform a formal sensitivity analysis comparing individuals who slept alone versus with a partner. Indeed, Ravindran et al. reported that, while the Withings Sleep Analyzer provided similar nocturnal sleep assessment performance when compared to an actigraphy wrist sensor with sleep diary in-home, under controlled conditions, the Withings Sleep Analyzer was less accurate^45^. This highlights that while these sensors offer valuable longitudinal sleep data in real-world settings, the presence of bed partners and other environmental factors can introduce measurement noise that should be considered when interpreting results. As such, even with sensors positioned on the participant’s side of the bed, residual effects from bed partners, particularly those with sleep disorders such as sleep apnoea, cannot be entirely ruled out. Finally, in using under-the-mattress sensors, we must consider several other potential confounders. For example, mobility impairments may reduce movement, which may be misinterpreted as better sleep, and nocturnal agitation or medications may alter sleep patterns. These limitations should be taken into account when interpreting the Sleep Age Index and other derived sleep metrics.

Several limitations also arise from the underlying data. In Supplementary Information Section S9, we discuss the model reliability and calibration and find that, while our model is reasonably well calibrated, it still experiences some non-linear miscalibration when predicting dementia. This motivates the application of our stratification thresholds to the model’s dementia risk scores, as it allows us to balance model accuracy with the number of ‘Red’ flags; however, in deployment, the trade-off between sensitivity and specificity should be re-evaluated to ensure the groups remain well-calibrated for the cohort being studied. Considering sex (see Supplementary Information Table S9), we see that while our age estimation model had similar performance for both sexes, female participants were more likely to be predicted as having dementia (see Supplementary Information Table S11). Considering age (see Supplementary Information Table S12 in Supplementary Information Section S11 for a preliminary analysis of model performance across each age group), we see that our model performs well between the ages of 50–59 and on participants over 80, reasonably well between the ages of 60–69, but has a tendency to yield false negatives between the ages of 70–79, while we also note a large imbalance in the number of samples from each cohort within the age ranges of 50–59 and 80+, respectively. When deploying this as a risk screening tool, the proportion of labels might impact these metrics. For this proof-of-concept study, we have trained a single risk prediction model to prevent overfitting in the model due to insufficient data; however, another direction of this work could look to train separate models to make predictions on each of the four age bands, as well as for male and female participants.

A notable limitation of the current study is independently scaling external datasets for age estimation, which was necessitated by changes in hardware and firmware across data collection periods (see Supplementary Information Section S5). While this approach was appropriate for the purposes of this preliminary study, it is not directly applicable to individual-level clinical interpretation, where group-level normalisation parameters would not be available prior to assessment. We anticipate that a prospective validation study, with consistent hardware, firmware and data collection protocols throughout, would eliminate the need for independent scaling, allowing a single unified scaling approach to be applied and improving portability and clinical applicability. Future work in this field should look to formally characterise the degree of drift attributable to firmware and environmental differences.

Seasonality represents another potential contributor to this paradox. In Supplementary Information Section S12, we present an evaluation of the effect of seasonality on both our age estimation and our risk prediction. While there was no meaningful effect on the final classification decisions, in both the Population Control and Dementia cohorts, differences in SAI were observed between the Winter and Summer months. Notably our control dataset lacks substantial representation from Spring months. As such, its contribution to the observed SAI paradox warrants further investigation in larger, seasonally balanced datasets. Further work would benefit from examining the potential impact of environmental factors such as temperature and daylight hours on sleep metrics. Moreover, validating this framework in regions with different seasonal patterns might further improve generalisation and robustness.

Our prediction model did not include information on comorbidities or medications, factors which may influence the risk of dementia. In particular, psychoactive medications, including sedatives, hypnotics, and stimulants, may modulate cortical excitability and cholinergic tone and could therefore influence both individual sleep-stage architecture and SAI measurements. We recommend that future studies with larger samples systematically record and control for such medications to better isolate the neurophysiological signal of interest. Multimorbidity is also associated with a higher prevalence of sleep problems, increased nocturnal disturbances and altered sleep-wake routines^15–17^. The motivation for this work was to develop a framework for identifying high-risk, clinically complex populations for dementia. A future direction of this work is to incorporate these variables to tailor the model, thereby more accurately differentiating between individuals with and without dementia, as well as in the context of other conditions and treatments, enhancing predictive performance and clinical relevance.

Here, our age estimation model is trained on data from the general population, which allows us to measure deviations in sleep patterns between people living with dementia and the general population and identify differences associated with dementia. This is central to the metric’s interpretability. However, future work could explore incorporating PLWD data during validation and calibration stages, rather than training, to better characterise how pathological sleep signatures map onto the learned age-feature space and whether this resolves the observed paradox.

Our findings invite comparison with other sleep-based chronological versus cognitive age assessment methods. EEG and PSG-based ‘brain-age’ metrics have demonstrated sensitivity to neurodegenerative pathology but are episodic in nature and typically limited to single or few-night assessments in clinical settings limiting scalability. EEG-based ‘brain-age’ metrics in the current literature shows older predicted ages in populations with dementia^27^, which is biologically intuitive. The contrasting finding of younger SAI in our Dementia and Pilot High-risk cohorts therefore warrants specific consideration. This divergence likely reflects fundamental differences in what each modality captures. EEG/PSG directly index cortical and polysomnographic neural activity, whereas actigraphy-derived metrics reflect broader behavioural and autonomic sleep patterns. This divergence suggests that SAI and EEG/PSG-based metrics may capture complementary, non-overlapping aspects of neurodegenerative pathology, and future work should formally examine their relationship. We propose that SAI, derived from minimally obtrusive consumer devices, occupies a complementary role, offering accessible, longitudinal monitoring capability.

In conclusion, we provide preliminary evidence for a scalable framework to identify the risk of dementia in the general population, which, with further testing, has the potential to transform the field of dementia care. To the best of our knowledge, while previous studies have used EEG sleep data to predict dementia directly^51^, this is the first study that uses remote sleep monitoring data to predict dementia, using SAI as a proxy measure for cognitive health. The proposed pipeline takes advantage of the inherent interpretability of machine learning models to develop a transparent and explainable framework, designed to enhance clinical decision-making. In addition, utilising stratification techniques enables us to calibrate our classification model for improved performance in clinical settings. Furthermore, SHAP analysis (such as in Fig. 2 presents explainable results that allow researchers and clinicians to explore why the algorithm has made a particular prediction and better understand the difference between how ‘normal’ ageing and dementia affect sleep. By identifying sleep patterns associated with cognitive decline from those associated with typical ageing, our framework can be used to guide discovery research. Finally, we conducted a prospective pilot study on a high-risk population of multi-morbid individuals to determine the model’s usefulness as a tool for predicting dementia risk.

This approach can be scaled rapidly. The use of this framework has the potential to markedly improve the quality of life for PLWD, supporting earlier diagnosis and the development of more timely care plans.

## Methods

### Data use and study design

We include sleep data collected from participants across three study cohorts (each with a different set of study objectives and population cohort) using an under-the-mattress sleep sensor: a dataset obtained from users of the Withings sleep mat monitoring device within the general population, referred to in this paper as the Population Control cohort; a dataset obtained from the Minder Integrated Health Management Study (see https://mindermeetingplace.com), the UK Dementia Research Institute (UK DRI) Care Research & Technology (CRT) (see https://www.ukdri.ac.uk/centres/care-research-technology) Centre’s ongoing longitudinal study of PLWD in the United Kingdom, which began in 2018, referred to in this paper as the Dementia cohort; and a dataset obtained via the Resilient study (see https://www.sabp.nhs.uk/research/current-studies/research-everyone/resilient-1and https://www.hra.nhs.uk/planning-and-improving-research/application-summaries/research-summaries/resilient-2/), the Surrey and Borders Partnership (SABP) NHS Foundation Trust’s ongoing feasibility study, which began in 2023, collecting in-home monitoring data from a cohort of older adults (65+) diagnosed with multi-morbidity at the risk of cognitive decline and their study partners (who provide essential care and support at home of the main study participants), referred to in this paper as the Pilot High-risk cohort.

The Population Control cohort comprises individuals from the general population using the Withings sleep mat monitoring device (see https://www.withings.com/uk/en/sleep-analyzer). This dataset includes over 1 million nights of sleep from June 2020 to March 2021 across 5574 participants in Europe.

Eligible study participants for the Minder Health Management Study (the Dementia Cohort) include adults older than 50 years of age who are clinically diagnosed with dementia, mild cognitive impairment (MCI), frailty, stroke, or traumatic brain injury. Individuals with severe psychiatric symptoms (including the presence of active suicidal thoughts or severe mental health conditions such as depression, anxiety, psychosis, and agitation), severe sensory impairment, those enroled in clinical trials who were in receipt of any investigational drug within 30-days prior to being enroled in the Minder study, or those receiving treatment for terminal illness are excluded from the study (see https://www.isrctn.com/ISRCTN71000991). A more comprehensive list of inclusion and criteria can be found here: https://www.isrctn.com/ISRCTN71000991. This study was conducted in collaboration with Imperial College London and the SABP NHS Foundation Trust. Participants were recruited through multiple NHS and social care channels, including primary care networks, urgent and emergency services, older adult mental health teams, and specialist memory services. Participant diagnoses were obtained from their electronic health records at baseline. Diagnoses were made by clinicians, typically within community settings or memory clinics. Following an initial referral from the participant’s GP to a community memory clinic, a detailed history is gathered from the patient and their family regarding behavioural, functional, memory, and everyday life changes, including the ability to perform daily tasks. Psychological and cognitive assessments are usually conducted alongside physical and neurological examinations and brain imaging. This collective information is used to determine an appropriate diagnosis, recorded in the patient’s medical record and officially documented in a clinic letter from the diagnosing physician. While processes differ slightly among clinicians, most utilise the MMSE or MoCA for dementia diagnoses, with none using the BADL. These diagnoses are the formal baseline diagnoses in this study. Throughout the study, participants undergo additional neuropsychiatric assessments and brain imaging at regular intervals. An internal multidisciplinary team comprising psychiatrists, radiologists, and neurologists reviews changes in individual diagnoses based on symptom progression since baseline. If a diagnosis change is agreed for the participant, this is recorded as the preferred diagnosis, superseding the initial baseline diagnosis established from the initial assessment in community memory clinics. Such changes are supported either by the natural progression (e.g., from MCI to Alzheimer’s Disease) or by an alternative diagnosis based on new evidence and more thorough investigation that may not have been available initially. This dataset includes over 110,000 nights of person-data across 160 participants collected from the 1st of December 2018 to the 3rd of April 2025.

Eligible study participants for the Resilient study (the Pilot High-risk cohort) include individuals aged 65 or older with a clinical diagnosis of at least two long-term health conditions associated with an increased risk of dementia. These included, but were not limited to, arthritis, chronic kidney disease, heart disease or heart failure, depression and other mental health disorders, diabetes, hypertension, liver disease, and stroke. Participation was open to individuals with or without a study partner. Exclusion criteria include severe mental health conditions (such as psychosis, severe depression, agitation, or anxiety), profound sensory impairments, current treatment for a terminal illness with a life expectancy of less than six months or in the final year of life, and the inability to provide informed consent. Eligibility was determined by the principal investigator in accordance with the predefined inclusion and exclusion criteria outlined here and in the study protocol. This dataset includes over 14,000 nights of person-data collected between the 25th of August 2023 to the 25th of February 2025 across 74 participants and their study partners (55 participants, 19 study partners).

### Ethical approval

The Population Control dataset was provided by Withings (https://www.withings.com) under a data-sharing agreement for research with Imperial College London. Withings is certified in ISO 27001 and Health Data Hosting (HDS) which comply with the same level of security as health professionals. Users of the Withings under-the-mattress sleep sensor consented to their data being used as part of the Withings research programme (see https://www.withings.com/eu/en/research?srsltid=AfmBOoooTHreAd0rFxCqiX5XShZzS7-IUeZlKopv5uNJD3euOpSssDGs) and may withdraw their consent at any time. The data used in this work was fully anonymised before sharing, so re-consent was not required. Withings data collection is in compliance with the GDPR, with their data privacy policy (https://www.withings.com/uk/en/legal/privacy-policy) citing “legitimate interest” as one of the lawful biases for processing personal data, in this case, Withings processes non-identifying data, to improve research. Information governance and impact assessment approval for storing and processing data shared by Withings was obtained from the Surrey and Borders Partnership NHS Foundation Trust (reference number: 20201009-DRI).

The Minder Health Management Study is registered with the National Institute for Health and Care Research (NIHR) in the United Kingdom under the Integrated Research Application System (IRAS) registration number 257561 and received ethical approval from the London-Surrey Borders Research Ethics Committee; TIHM 1.5 REC: 19/LO/0102. Upon joining the study, all participants and their study partners provided written informed consent to participate in the study and for their data to contribute to publications. If deemed unable to do so, consent was obtained from either a participant’s legal representative(s) or power of attorney holder(s) after discussions with family members and clinicians to ensure that participation in this study was in the participant’s best interest. Approval to collect data from the under-the-mattress sleep sensor in the Minder Health Management Study was granted by a clinical IRB panel.

The Resilient study received ethical approval from the London-Surrey Borders Research Ethics Committee and the Health Research Authority, and was formally registered on the Integrated Research Application System (IRAS) under reference number 321104. All participants provided written informed consent prior to enrolment. Participants received a participant information sheet that explained how their personal data would be collected, used and stored in compliance with the General Data Protection Regulation (GDPR). Individuals who were deemed to lack capacity at baseline were excluded from the study.

For both the Minder Health Management Study and the Resilient study, capacity to consent was assessed in accordance with Good Clinical Practice (GCP) (see https://www.nihr.ac.uk/career-development/clinical-research-courses-and-support/good-clinical-practice), as outlined in the UK Policy Framework for Health and Social Care Research (see https://www.hra.nhs.uk/planning-and-improving-research/policies-standards-legislation/uk-policy-framework-health-social-care-research/) and the Mental Capacity Act 2005 (see https://www.legislation.gov.uk/ukpga/2005/9/contents).

### Data pre-processing procedures and characteristics

To control for age and ensure a more meaningful comparison between datasets, we select only those individuals aged 50 years and older from the Population Control dataset. All datasets underwent identical pre-processing. Aggregated by participant, data was cleaned, and missing dates were imputed using a rolling mean with a 7-day window in which a minimum of 5 days needed to be non-zero values. All remaining dates with missing data were removed. To maximise the information in the data, we generated two additional features (‘Time to Bed (angles)’ and ‘Time to Rise (angles)’). The dataset was comprised of a total of 16 features (‘Time to Bed (angles)’, ‘Time to Rise (angles)’, ‘Time in Bed (s)’, ‘Time Out of Bed (s)’, ‘Bed Time Period (s)’ (total time in bed), ‘Rapid eye movement (REM) Duration (s)’, ‘Light Duration (s)’, ‘Deep Duration (s)’, ‘Awake Duration (s)’, ‘Time Snoring (s)’, ‘Heart rate (HR) Average (beats per minute or BPM)’, ‘HR Minimum (BPM)’, ‘HR Maximum (BPM)’, ‘Respiratory rate (RR) Average (breaths per minute or BPM)’, ‘RR Minimum (BPM)’, ‘RR Maximum (BPM)’). For the purposes of training our age estimation model, we grouped participants into one of four age bands (50-59, 60-69, 70-79, 80+) based on their chronological age. We then filtered for participants who did not have at least *n* = 30 days of consecutive-day data. We then derived *n* = 30-day arrays of data for each participant using a rolling window of 10 days. This approach allowed us to maximise data usage, minimise information loss between data periods, and improve our ability to detect more gradual changes. Our choice to select *n* = 30 days based on previous work by this group^35^ was further validated during preliminary evaluations of our age estimation models (see Supplementary Information Section S6). We calculate statistics (the mean, median, minimum, maximum, standard deviation and interquartile range of each feature) for each array of participant data, capturing complementary perspectives of the data (e.g. the mean captures the average effect of a variable’s distribution, while the median is more robust to skewness and outliers). Sex is included as a confounding variable, with the data transformed into a numeric format using one-hot encoding. The resulting processed data was comprised of a total of 98 features.

Demographics of the three datasets are provided in Table 1, reporting both the original dataset demographics and those of the processed versions used in this study. Male and female assignments were self-declared by participants. We also include information on the primary diagnoses and Mini-Mental State Examination (MMSE) scores used to assess the severity of cognitive impairment for the Minder Health Management Study participants on entry into the clinical study (see Tables S1 and S2 in Supplementary Information Section S2). Additionally, we include the top five most common diagnoses and medications for participants in the Resilient study (see Tables S3 and S4 in Supplementary Information Section S2, respectively), as well as ACE-III scores used to assess the severity of cognitive impairment on entry into the clinical study, along with the respective severity classifications^52,53^ (see Table S5 in Supplementary Information Section S2). Additionally, we report the PHQ-9 and GAD-7 scores, as well as the respective severity classifications of depression and anxiety^54,55^ for study participants at the time of entry into the clinical study (see Tables S6 and S7 in Supplementary Information Section S2).

### Analysis platform

All analyses were performed on a secure computing environment at Imperial College London in Python version 3.13.2 environment. The Pandas^56^ (version 2.2.3), NumPy^57^ (version 2.1.3), SciPy^58^ (version 1.15.2), statsmodels^59^ (version 0.14.4), and Scikit-Learn^60^ (version 1.6.1) packages formed much of our pipeline. All visualisations were plotted using Matplotlib^61^ (version 3.10.1), Seaborn^62^ version (0.13.2), pyCompare^63^ version (1.5.4), and SHAP (SHapley Additivie exPlanations), including the high-speed Tree Explainer algorithm^64,65^ (version 0.47.0).

### Statistical Analyses

Prior to deriving our data arrays for age estimation, we performed statistical analyses on individuals from the Population Control cohort (the processed dataset) with at least 30 consecutive days of data. We conducted between-subjects parametric (analysis of variance, or ANOVA) and non-parametric (Kruskall-Wallis) tests to investigate the relationship between age group and nocturnal activity and physiological data. We modelled the fixed effects of age. Post-hoc, Welch t-tests were used to identify which groups differed from each other. Holm-Bonferroni was used to correct for multiple comparisons. See Fig. S2 in Supplementary Information Section S4 for a visualisation of these comparisons. These preliminary analyses verified the validity of using these features to predict sleep age. After estimating the sleep age, we first calculated the Cohen’s d statistic to measure model performance between sexes for both the Population Control and Dementia cohorts. We then conducted a between-subjects Welch’s t-test (parametric) to investigate the relationship between the cohort to which the individual belonged and the SAI. We also used a one-way between-subjects ANOVA to investigate the relationship between severity of cognitive impairment and SAI, followed up by post-hoc Tukey’s HSD (Honestly Significant Difference) tests. Finally, we used a one-way between-subjects ANOVA to investigate the effect of seasonality on both our age estimation and dementia risk prediction models, followed up by post-hoc Tukey’s HSD tests where necessary. Unless otherwise stated, we use standard significance reporting (*p* < 0.0001 denoted as ***p < 0.001 denoted as **p < 0.05 denoted as *, and *p* < 0.1 denoted as ⋅ ). For our pilot study, we conducted a Bland-Altman analysis^34^ to measure the strength of agreement between our model’s predictions and the ground truths established by dementia experts in the Pilot High-risk cohort. Then, using the statsmodels^59^ package (version 0.14.4), we also conducted linear mixed effects modelling to examine the relationship between the adjusted risk scores and measures of cognitive impairment (ACE-III), depression (PHQ-9) and anxiety (GAD-7) in the Pilot High-risk cohort as the respective outcome variables. We also accounted for within-subject variability by specifying random intercepts for each participant.

### Framework for age estimation

Our age estimation models were trained and evaluated on the processed Population Control dataset (*n* = 1567 participants assumed to be representative of typical ageing). To ensure generalisability, this dataset was split into training and testing subsets in an approximate 80:20 ratio, split by ID, with stratification applied in the four age bands to preserve age distribution across the training and test sets. The training data represented 80% of the participant IDs (*n* = 1253 participants) and the testing data represented the remaining 20% (*n* = 314 participants). During model development and whilst optimising model hyperparameters, validation sets were produced by splitting the training data (by participant ID) using stratified k-fold cross-validation. See Supplementary Information Fig. S1 ‘Age estimation’ in Supplementary Information Section S1 for a visualisation of this evaluation. Stratification ensured that each fold contained a balanced representation of each age group compared to the whole training dataset, which is particularly important here due to the imbalance in the age group distributions. Finally, we used mean absolute error, mean squared error, and balanced mean absolute error to measure model performance (for definitions of metrics, see Supplementary Information Section S3).

For predicting sleep age, we tested three commonly used machine learning models for time-series regression: Elastic Net^66^, Huber^67^, and XGBoost^68^. These models represent a diverse and robust mixture of models by which to benchmark performance and get insights into feature importance. Hyperparameter optimisation was performed using k-fold cross-validation (k = 10), with the model producing the lowest mean absolute error on validation data selected for the final analysis. See Supplementary Information Fig. S1 ‘Age estimation’ in Supplementary Information Section S1 for a visualisation of this evaluation. Supplementary Information Section S5 contains information on the decisions made regarding each step of the regression pipeline. Furthermore, given the imbalanced distribution of ages in the Population Control dataset, we used this evaluation to explore sample weighting as a strategy to mitigate potential bias towards overrepresented ages. We applied sample weighting using the discretised age groups, assigning weights inversely proportional to bin frequency.

We initially selected *n* = 30 days to pre-process our datasets, and we used this validation technique to evaluate model performance using statistics derived from 30, 60 and 90-day arrays of data, respectively. While statistics derived from 90-day arrays often provided slightly better performance across models, with and without sample weighting, (see Table S8 in Supplementary Information Section S6), the improvement was too marginal compared to the prediction delay. 30-day windows allow for more frequent and timely updates, key for clinical monitoring. Moreover, using a shorter time window increases the number of usable data points, improving the robustness and generalisability of the model, with the 30-day window resulting in nearly 185% more samples, helping to reduce overfitting (see Fig. S3 in Supplementary Information Section S5). The selection of *n* = 30 days resulted in the final sample and participant numbers for the processed datasets as presented in this paper.

### Framework for predictive analysis

Our risk prediction model was trained and evaluated on a dataset of the learned SAI from both the held-out test set of the Population Control dataset and the Dementia cohort. To allow for research interpretability and to improve model performance and generalisability, we also examined the impact of using sex and a sex-feature interaction. We trained a binary classification task to distinguish individuals in the Dementia cohort from those in the Population Control. We performed an ID split to generate training and testing subsets in an approximate 80:20 ratio, stratified by cohort. The training data represented 80% of the total data (*n* = 334 participants) and the testing data represented the remaining 20% (*n* = 85 participants). During hyperparameter and threshold optimisation, validation sets were produced by splitting the training data (by participant ID) using stratified k-fold cross-validation. See Fig. S1 ‘Dementia risk prediction’ in Supplementary Information Section S1 for a visualisation of this evaluation. We used sensitivity, specificity, and the area under the precision-recall curve to measure model performance (for definitions of metrics, please see Supplementary Information Section S3).

We tested two commonly used classification models: Logistic Regression^69^ and XGBoost^68^. These models act as effective baselines representing a contrast between linear and non-linear approaches, respectively, with Logistic Regression offering interpretability, while XGBoost provides a more powerful model capable of capturing complex feature interactions. Hyperparameter optimisation was performed using k-fold cross-validation (k = 10), with the model producing the highest mean precision-recall area under the curve score on validation data selected for the final analysis. See Supplementary Information Fig. S1 ‘Dementia risk prediction’ in Supplementary Information Section S1 for a visualisation of this evaluation. Supplementary Information Section S5 contains information on the decisions made regarding each step of the classification pipeline. While there was only a slight imbalance in the distribution of individuals from each cohort in the dataset, we once again used this evaluation to explore sample weighting as a strategy to mitigate potential bias towards the overrepresented class, assigning weights inversely proportional to the cohort frequency.

### Stratification of risk scores for clinical reporting

We extracted probability estimates from the model as a measure of dementia risk. These risk scores were then stratified into three groups (Green, Amber, and Red), denoting low, medium, and high risk of dementia, respectively. These groupings provide a systematic framework to inform clinical decision-making, offering interpretable and data-driven insights. Varying these thresholds allows us to balance sensitivity and specificity with model uncertainty, which can be used to reveal the key difference between individuals the model predicted as being at high and low risk of dementia. This approach was first developed in our group by Capstick et al. and has since been used across various research scenarios^70–72^. Supplementary Information Section S10 provides further information on the process of stratification.

The optimal thresholds used in our final analysis were calculated using k-fold cross-validation (k=5), using the model’s predictions of the validation data after being re-trained with the best hyperparameters on the training data. By choosing 5-fold cross-validation, we limit the amount of model fine-tuning, avoid overfitting to the validation data. See Fig. S1 ‘Dementia risk prediction’ in Supplementary Information Section S1 for a visualisation of this evaluation.

#### CR&T group

Acknowledgement list for UK Dementia Research Institute (UK DRI) Care Research & Technology (CR&T) Centre publications using the Minder core data set. The primary contact for this group is: minder-enquiries@imperial.ac.uk.

**Leadership and Management:** David Sharp (Director), Danielle Wilson (Centre and Research Commercialisation Manager), Sarah Daniels (Health and Social Care Lead), Ramin Nilforooshan (Clinical Lead), David Wingfield (General Practice Lead), Matthew Harrison (Human-Centred Design Lead), Shlomi Haar (Movement Data and Living Lab Lead), Nora Joby (Data Science Lead), Mara Golemme (Scientific Project Manager), Stephanie Lietz (Scientific Project Manager), Margherita Tecilla (Scientific Project Manager).

**Behaviour and Cognition Group:** David Sharp (Group Lead), Michael David, Martina Del Giovane, Neil Graham, Magdalena Kolanko, Helen Lai, Lucia M Li, Mark Crook Rumsey, Emma Jane Mallas, Alina-Irina Serban, Eyal Soreq, Abidemi Otaiku, Megan Parkinson, Thomas Parker, Success Fabusoro, Emily Beal, Julian Jeyasingh Jacob, Gaia Frigerio, Anastasia Mirza-Davies, Ethan de Villiers.

**Bioelectronic Systems Group:** Timothy Constandinou (Group Lead), Alan Bannon, Danilo Mandic, Ziwei Chen, Charalambos Hadjipanayi, Ghena Hammour, Bryan Hsieh, Amir Nassibi, Adrien Rapeaux, Ian Williams, Maowen Yin, Niro Yogendran.

**Robotics and AI Interfaces Group:** Ravi Vaidyanathan (Group Lead), Maria Lima, Ting Su, Melanie Jouaiti, Maitreyee Wairagkar, Carlos Sebastian Castillo, Panipat Wattansiri, Thomas Martineau, Mayue Shi, Tianbo Xu, Alejandro Valdunciel, Reneira Seeamber, Annika Guez, Zehao Liu, Saksham Dhawan, Alina-Irina Serban.

**Translational Machine Intelligence Group:** Payam Barnaghi (Group Lead), Nan Fletcher-Lloyd, Samaneh Kouchaki, Alexander Capstick, Chloe Walsh, Louise Rigny, Marirena Bafaloukou, Jin Cui, Yu Chen, Nivedita Bijlani, Iona Biggart, Antigone Fogel, Nathalia Cespedez, Zeinab Ghannam.

**Point of Care Diagnostics Group:** Paul Freemont (Group Lead), Michael Crone, Kirsten Jensen, Martin Tran, Thomas Adam, Raphaella Jackson, Alexander Webb, David Wingfield.

**Sleep and Circadian Group:** Derk Jan Dijk (Group Lead), Anne Skeldon, Kevin Wells, Ullrich Bartsch, Ciro Della Monica, Kiran KG Ravindran, Victoria L Revell, Hana Hassanin, James Woolley, Iris Wood-Campar, Sarmad Al Gawwam, Aravind Kumar Kamaraj, Marta Pineda Messina.

**Brain and Movement Group:** Shlomi Haar (Group Lead), Nathan Steadman, Federico Nardi, Cosima Graef, Alena Kutuzova, Assaf Touboul, Nicolas Calvo Peiro, Jenna Yun, Sean Carr, Uri Rosenblum-Belzer, Emma Burroughs.

**Computational Neurology Group:** Gregory Scott (Group Lead), Adela Desowska, Anastasia Gailly de Taurines, Ruxandra Mihai, Nina Moutonnet.

**Human Centred Design Group:** Matthew Harrison (Group Lead), Sophie Horrocks, Brian Quan, Victoria Simpson.

**Site Investigators and Key Personnel:**

**Surrey and Borders Partnership NHS Foundation Trust:** Ramin Nilforooshan (Chief Investigator), Jessica True (Research and Development Manager), Olga Balazikova (Research and Development Manager), Behnam Shariati (Research Doctor), Chloe Walsh (Research Co-ordinator), Nicole Whitethread, Matthew Purnell, Vaiva Zarombaite, Lucy Copps, Olivia Knight, Gaganpreet Bangar, Sumit Dey, Chelsea Mukonda, Jessica Hine, Luke Mallon, Saijal Jhala, Oliver Sargentoni, Amy Alves, Mahan Heydari, Alexandra Cairns (Clinical Monitoring Team).

**Hammersmith and Fulham Partnership** David Wingfield (Principal Investigator), Monica Morim (Research Nurse/Paramedic), Anesha Patel, Ruby Lyall, Sanara Raza, Success Fabusoro, Gaia Frigerio, Maria Rasulo, Catalina Chavarro Novoa, Martynas Stonkus, Prital Patel, Zara Prem (Clinical Studies), Naomi Hassim, Pippa Kirby (Research Allied Healthcare Professionals) John Patterson(London Borough of Hammersmith and Fulham Support: Assistive Technology), Mike Law (Business Development), Andy Kenny (Social Services).

**Minder Digitally Enabled Care for Dementia (MinderCare)** David Sharp (Principal Investigator), James Bird (Co-Investigator), Sarah Pearse (Co-Investigator), Joanna James, Janibo Amade Cassimo, Aglaja Dar, Pandora Wright, Lucia Li, Anastasia Mirza-Davies, Julian Jeyasingh Jacobs, Zinca Zecevic, Sarah Daniels, David Wingfield (Clinical Team), Success Fabusoro, Gaia Frigerio, Maria Rasulo, Catalina Chavarro Novoa (Research Technicians).

## Supplementary information

Supplementary Information

Supplementary Data 1

## Data Availability

The Withings dataset was provided under a data-sharing agreement for research with Imperial College London and is not publicly available. A subset of the Minder dataset has been made publicly available and can be found on Zenodo at: https://zenodo.org/records/7622128^73^. A full description of this data subset is published in Nature Scientific Data and can be found here: https://doi.org/10.1038/s41597-023-02519-y^74^. The extended Minder dataset is available from the corresponding authors upon reasonable request. The Resilient dataset has been made publicly available and can be found on Zenodo at: https://zenodo.org/records/16755408^75^.

## Code availability

To promote the sharing of resources, the models presented in this paper, as well as all associated code, can be found on GitHub at https://github.com/tmi-lab/Sleep-Age-Dementia. The code for experiments presented in this study will be made available by the corresponding author upon reasonable request.
